# 3D Printing of Fiber-Reinforced Plastic Composites Using Fused Deposition Modeling: A Status Review

**DOI:** 10.3390/ma14164520

**Published:** 2021-08-12

**Authors:** Salman Pervaiz, Taimur Ali Qureshi, Ghanim Kashwani, Sathish Kannan

**Affiliations:** 1 Department of Mechanical and Industrial Engineering, Rochester Institute of Technology, Dubai Campus, Dubai P.O. Box 341055, United Arab Emirates; taq3950@rit.edu; 2 Engineering Division, New York University Abu Dhabi, Abu Dhabi P.O. Box 129188, United Arab Emirates; gak289@nyu.edu; 3 Department of Mechanical Engineering, American University of Sharjah, Sharjah P.O. Box 26666, United Arab Emirates; skannan@aus.edu

**Keywords:** FRP, 3D printing, defense, FDM

## Abstract

Composite materials are a combination of two or more types of materials used to enhance the mechanical and structural properties of engineering products. When fibers are mixed in the polymeric matrix, the composite material is known as fiber-reinforced polymer (FRP). FRP materials are widely used in structural applications related to defense, automotive, aerospace, and sports-based industries. These materials are used in producing lightweight components with high tensile strength and rigidity. The fiber component in fiber-reinforced polymers provides the desired strength-to-weight ratio; however, the polymer portion costs less, and the process of making the matrix is quite straightforward. There is a high demand in industrial sectors, such as defense and military, aerospace, automotive, biomedical and sports, to manufacture these fiber-reinforced polymers using 3D printing and additive manufacturing technologies. FRP composites are used in diversified applications such as military vehicles, shelters, war fighting safety equipment, fighter aircrafts, naval ships, and submarine structures. Techniques to fabricate composite materials, degrade the weight-to-strength ratio and the tensile strength of the components, and they can play a critical role towards the service life of the components. Fused deposition modeling (FDM) is a technique for 3D printing that allows layered fabrication of parts using thermoplastic composites. Complex shape and geometry with enhanced mechanical properties can be obtained using this technique. This paper highlights the limitations in the development of FRPs and challenges associated with their mechanical properties. The future prospects of carbon fiber (CF) and polymeric matrixes are also mentioned in this study. The study also highlights different areas requiring further investigation in FDM-assisted 3D printing. The available literature on FRP composites is focused only on describing the properties of the product and the potential applications for it. It has been observed that scientific knowledge has gaps when it comes to predicting the performance of FRP composite parts fabricated under 3D printing (FDM) techniques. The mechanical properties of 3D-printed FRPs were studied so that a correlation between the 3D printing method could be established. This review paper will be helpful for researchers, scientists, manufacturers, etc., working in the area of FDM-assisted 3D printing of FRPs.

## 1. Introduction

The properties of materials play a significant role in manufacturing of equipment for various industries, such as defense, automobile, aerospace, healthcare, and many similar sectors, with demanding applications. Fiber reinforced polymer (FRP) composites are a combination of fibers and matrixes that can be either thermoplastic, elastomer, or thermoset. The fiber provides a strength-to-weight ratio, the polymer composites cost less, and the process of making their matrix is quite easy. In 1960, FRP composites were the major structural component of the aerospace sector. In 1990–2006, FRP composites were the low cost and flexible solution for many manufacturing processes [1].

FRP composites have a high strength-to-weight ratio, good anti-wear properties and improved anti-aging capacities compared to traditional metal materials. FRP composites are light weight and provide high performance in many industries. The matrix in FRP composites mostly consists of thermosetting and thermoplastics. The thermosetting plastics in FRP are of an epoxy and polyurethane type and that of thermoplastics are polypropylene (PP), polyamide (PA), and polyetheretherketone (PEEK). FRP composites are also characterized by different fiber materials such as glass, carbon, aramid, and Kevlar. FRPs are also divided into subtypes by fiber length parameter such as short fibers (0.2–0.4 mm), and long fibers (10–25 mm). The method of forming and processing long and short CFRPs is by extrusion or injection molding. For the other type of continuous fiber-reinforced plastic, the processing is performed by winding, molding, impregnating, and pultrusion. The construction of continuous fiber-reinforced plastic components is a lengthy and complex process, and complex structure achievement is difficult because the viscosity is high for infusion during wet-out [2,3].

Additive manufacturing (AM)/rapid prototyping is said to be a method of integrating materials to make objects from computer aided design (CAD) models in consecutive films [4]. AM manufacturing provides low-cost, versatile products; thus, over the past years, the use of this technology to design improved products has become a large trend. Either with monolithic structures or micrometer solutions, AM is proving to beneficial [5]. The literature on FRPs focuses only on straight processes describing the properties of the product and the potential applications for it. Due to the complex interfacial adhesion in FDM printing of fiber-polymer composites, the performance of the component varies significantly, and there is a need to better understand its performance. The mechanical properties of 3D-printed carbon fiber-reinforced polymers are created so that the correlation between the types of additive manufacturing methods can be understandable. The techniques of combination composite materials impact on the weight-to-strength ratio as well as the tensile strength of the components and can play a critical role towards the service life of the components. Fused filament fabrication (FFF) is also a technique for 3D printing; it also allows for layered fabrication of parts using thermoplastic composites. Complex shape and geometry with enhanced mechanical properties can be obtained using this technique. 

**Figure 1:**
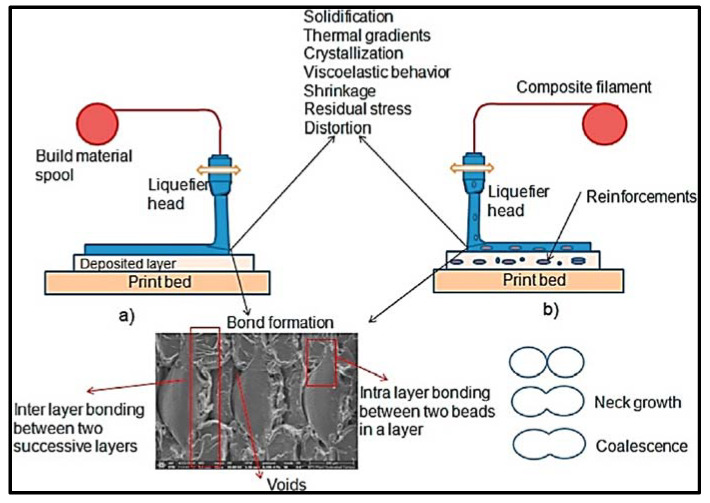
Schematic representation of the FDM process: (**a**) 3D printing of neat polymer; (**b**) 3D printing of polymer reinforced with particle fillers or short fibers [6] (reprinted with kind permission from Elsevier).

The literature [6,7,8,9,10,11,12] has revealed that the strength of FDM-printed parts is highly dependent on the printing phenomena and quality of the bond formation. Weak strength is associated with insufficient bond strength between the layers. It is also observed that the formation of bonds between layers is based on three phases, namely, surface contact, neck growth, and molecular diffusion [6]. It has been revealed that the second phase of neck growth is very important and can play a vital role towards the strength of FDM-printed parts. The quality of the bonds between layers is dependent on the size of neck formation and is controlled by molecular diffusion that happens between the polymeric chains at the interface. Gurrala and Regalla [13] also studied the coalescence of filament towards the strength of FDM-printed parts. In the study, it was revealed, by scanning electron microscopy, that neck growth was not uniform throughout the process, and at some locations there was no neck formation at all. The reason attributed to this observation was linked with the localized non-uniform cooling rates and temperature variations at different locations. Sun et al. [14] provide an in-depth study on the coalescence mechanism between filament layers. The study mentions the importance of phase 2, which facilitates adhesion and formation of molecular diffusion. The study also revealed that phase 2 is dependent on the contact angle between two filaments. Bonding occurs between adjacent layers of the filament and successive layers of the filament, known as intra-layers and inter-layers, respectively, as shown in the Figure 1 [6]. 

The review section of paper highlights the stated challenges in the development of carbon fiber-reinforced polymers and the challenges associated with its mechanical properties. The future prospects for the carbon fiber-reinforced polymers are also mentioned in this study, while it also puts a spotlight on areas requiring further investigation in rapid prototyping. The objective of this review paper was to corelate the mechanical properties of 3D-printed carbon fiber-reinforced polymers with respect to various 3D-printing techniques. 

## 2. Industrial Significance of FRP Composites 

**Figure 2:**
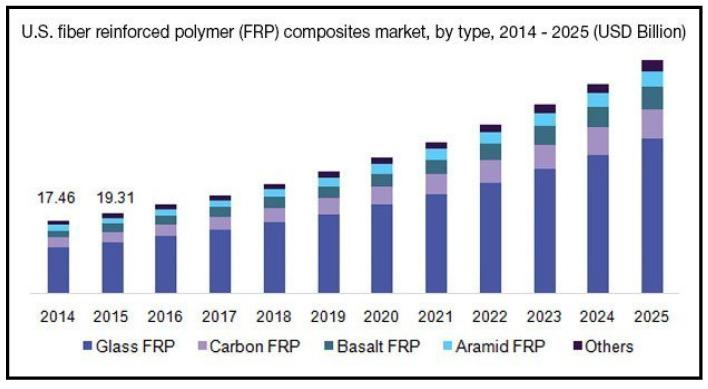
Forecast of the global growth of the FRP composite market, 2014–2025 [16].

**Figure 3:**
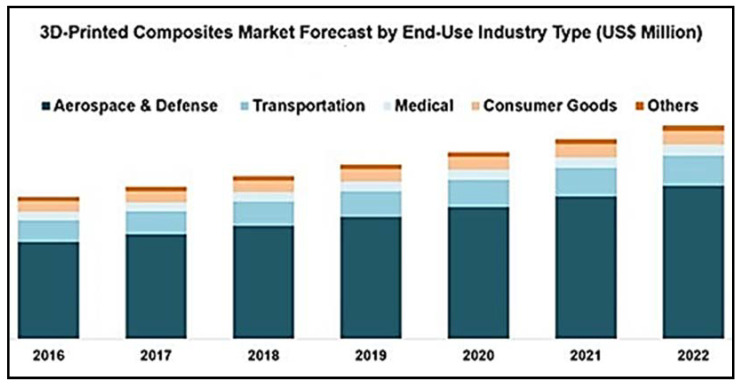
Forecast of the market growth of 3D-printed composites [17].

FRP provides the desired strength and stiffness while being light weight at the same time. This lightweight property is controlled by using the weight of the matrix in the fiber-matrix material system. In addition to these properties, FRPs are capable of operating at higher temperatures, chemical inertness, and have the ability to provide better damping [15]. Due to the fact of these qualities, FRP composites are rapidly replacing conventional ferrous and non-ferrous metals and their alloys. It can also be observed that the global market growth for FRP composites is increasing rapid. As reflected in Figure 2, the US composite market (FRP) is forecasted to have a compound annual growth rate of 11.3% from 2017 to 2025 [16]. At the same time, industry is adopting 3D-printing technologies to print FRP composites. As shown in Figure 3, the 3D-printed composites market is forecasted to grow to 111 million USD between 2017 and 2022 [17]. 

**Figure 4:**
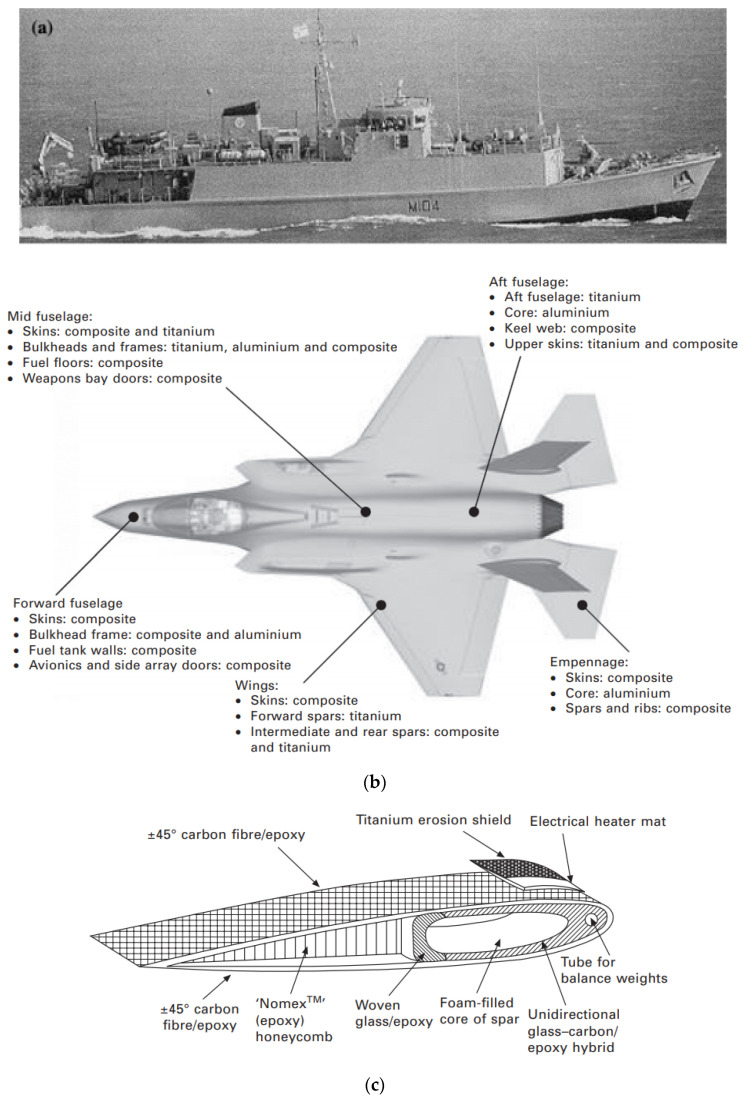
FRP composites in (**a**) Naval ships [19,20,21,22]; (**b**) an F-35 fighter jet [23]; (**c**) Helicopter blade [23] (reprinted with kind permission from Elsevier).

As shown in Figure 4, a visible increase in market growth can be noticed regarding 3D-printed FRP composite products with respect to the aerospace and defense sectors. In the below subsections, these sectors and related FRP applications will be discussed in detail. 

### 2.1. Defense and Military Sector

FRP composites are well suited for defense and military applications due to the fact of their high strength, light weight, corrosion resistance, and prototyping of complex geometries, etc. This class of materials was widely used in defense and military applications after World War II. They gained popularity over conventional metals and steels in the defense sector because of their anti-corrosiveness, fatigue resistance, and light weight. This was because structures experience excessive corrosion in salty seawater [18,19]. FRP composites are used in diverse applications such as military vehicles, shelters, war fighting safety equipment, fighter aircrafts, naval ships, and submarine structures. Figure 4 shows FRP composites in a submarine, an F-35 fighter jet, and a helicopter. These materials were favored due to the fact of their light weight and high strength, reliable performance, and ease in maintenance during service life. FRP composites are also utilized extensively in the protective clothing used by security enforcement bodies [20].

### 2.2. Aerospace Sector

**Figure 5:**
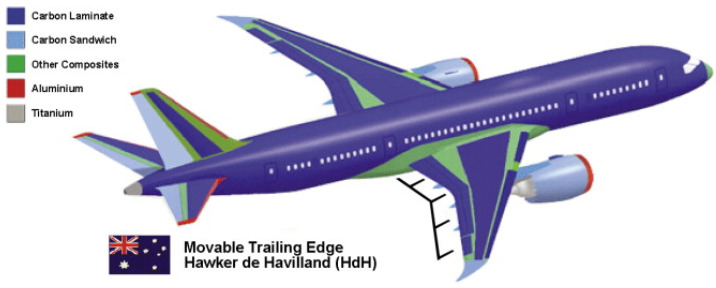
Material utilization in the Boeing 787 Dream Liner manufactured by HdH [24] (reprinted with kind permission from Elsevier).

FRP composites are widely used in the construction of passenger aircrafts. The Airbus A300 utilized CFRPs for spoilers, rudders, and airbrakes. Different famous FRP composites, such as CFRP, GFRP, and AFRP, were utilized in the Airbus A330/340 [15]. It is estimated that approximately 50% (by weight) of modern aircrafts are made of composites as shown below in the Boeing 787 Dream Liner in Figure 5. The Boeing 787 was the first air jetliner that utilized composite materials as leading structural materials in the airframe structure. The Boeing 787 carries 23 tons of composite materials. FRP composites are used in key body aircraft parts such as fuselage, upper and lower wing skins, radom, wing flaps, elevators, vertical fins, and horizontal stabilizer. Carbon-fiber epoxy is laid up with the help of robotic heads and fibers are reinforced in the desired directions to support maximum loads [21]. 

### 2.3. Automotive Sector

**Figure 6:**
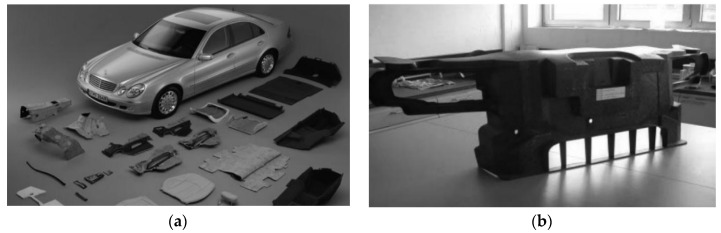
Natural composites applied in the automotive sector (**a**) Components of Mercedes-Benz E-Class (**b**) Ford Montagetrager front-end grill (available under a Creative Commons license [26]).

The Automotive sector was first to introduce better engineering materials to replace conventional metals and alloys to reduce cost and weight. It is reported that 75% of fuel consumption is directly linked to the weight of automobiles [25]. In addition, the automotive sector is highly competitive, and a higher performance with an extended service life is desired from different automotive components. Composite materials are used heavily in the load bearing and structural parts, such as the body, chassis, hoods, brakes, and electronics, of an automobile [20]. In the past, engine parts in the automobile sector were made from cast iron; the drawback of using cast iron was the reduced fuel efficiency and the speed of the engine was also slow. Now, cast iron parts are being replaced by aluminum alloys. A simple material cannot provide all the properties required for an effective product, so combinations of two or more materials were introduced to obtain the desired properties; these are known as composite materials [25]. Figure 6 shows some automotive parts constructed out of composite materials.

### 2.4. Construction Sector

**Figure 7:**
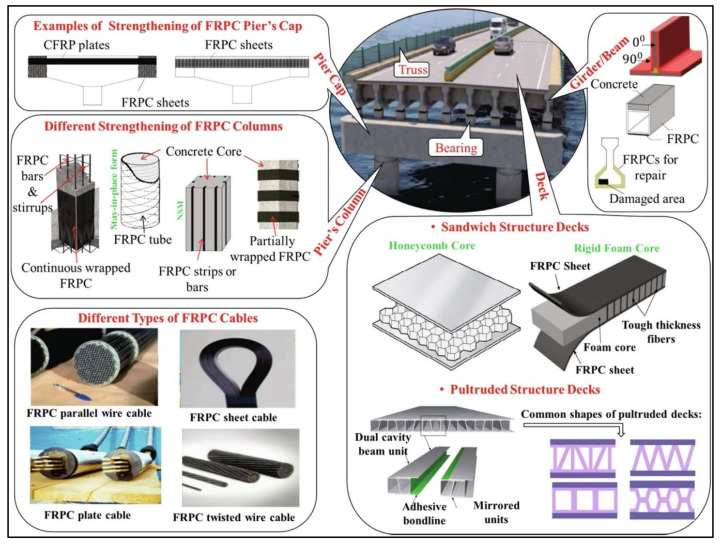
FRP utilization in bridge construction [27] (reprinted with kind permission from Elsevier).

FRP composites are utilized extensively in the construction sector. They have successfully replaced conventional steels, previously used to produce reinforcement bars for concrete constructions. In the construction sector, FRP composites are popular because of their low weight, high strength, corrosion resistance, high-impact strength, electromagnetic transparency, low operational cost, and low thermal conductivity. However, with respect to the construction industry, they also have limitations such as high brittleness, low shear and bending strength, vulnerability to fire, and high initial investment [1]. FRP composites are widely used in bridge construction. The major limitation associated with FRP composite curing is linked with exothermic resin. It releases heat during the curing process, and thicker samples restrict this heat that, in some cases, result in instant combustion. Many of the bridge structures being used all over the world are pultruded profiles generated from FRP composites. Figure 7 shows different ways in which FRP composite materials are used in bridge structures.

## 3. Fiber-Matrix Material System

**Figure 8:**
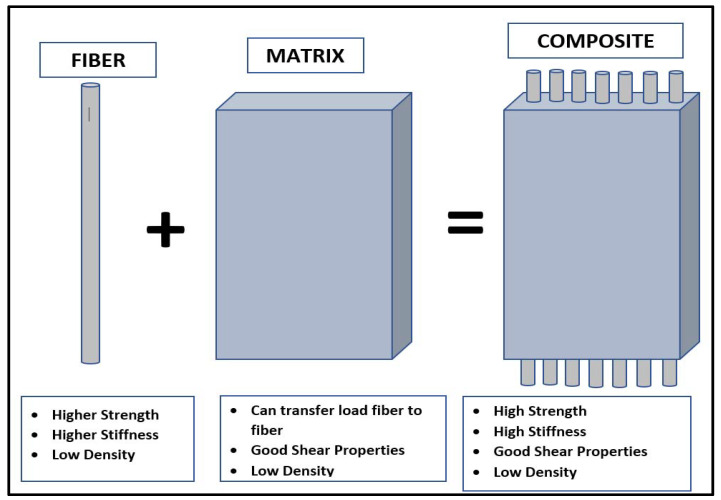
Schematic illustration of fiber-matrix mixture as composite material.

Composites are the materials that are made up of mixture of two or more distinct constituents. In order to be referred as composite materials, it is important that distinct constituents mix with each other at the macro level without any chemical interaction. In mixture form, the resulting product has properties significantly different from each constituent. Moreover, another important feature of composite materials is that one or more discontinuous constituent is embedded in a continuous constituent [28]. As per the nomenclature, the discontinuous constituents are called reinforcements and carry more strength and hardness, whereas the continuous constituent is referred to as the matrix and is generally weaker with respect to the reinforcements. Figure 8 represents schematic illustrations of the fiber-matrix material concept. Reinforcements can come in different geometric shapes and directly controls the strengthening. These reinforcements are classified as fiber or particle based. The resulting composites are referred to as fiber-reinforced composites or particulate-reinforced composites. 

As fiber reinforcements, there are many contender materials that provide beneficial results. Some famous fiber materials are silica, alumina, aramid, carbon, and boron. These fibers possess high strength but low plasticity. Fibers are selected to make FRP composites based on their specific gravity, strength, and modulus. Higher values of specific modulus and strength points to the fiber material being light weight with high strength and stiffness. Desired properties can be tailormade by introducing the correct fiber material, their volume fraction, and their orientation. 

**Figure 9:**
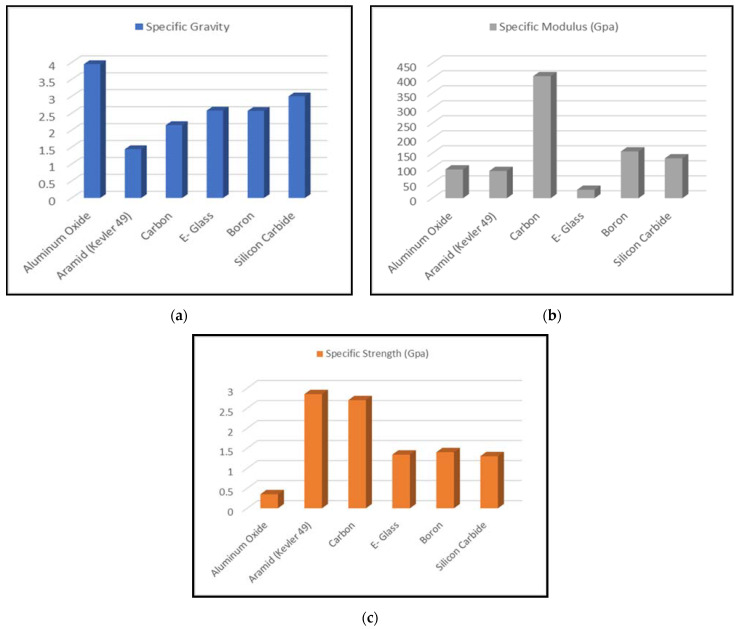
Comparison of different commonly used fiber materials: (**a**) specific gravity; (**b**) specific modulus (GPa); (**c**) specific strength (GPa) (based on data from [35]).

Matrix material is weaker in strength and shows relatively more plasticity with respect to the fiber materials. Matrix materials should be chemically and thermally compatible with the fiber materials. Matrix materials bind fiber materials and perform load transfer mechanism between fibers. Matrix material is selected based on the resulting FRP composite operating temperature. Different types of materials such as polymers, metals, and ceramics can be used to serve the matrix materials. In the case of FRP composites, matrix materials are generally polymers. There are two major types of matrix materials, namely, thermosetting plastics and thermoplastics [29]. The most commonly used thermosetting resins are polyester resin, vinyl-ester resin, and epoxy resin. Table 1 represents the most commonly used matrix-fibers FRP composites. However, polymers as matrix material also contain several flaws such as low operating temperature, high thermal expansion coefficient, interaction with moisture, and interaction with radiation [30,31,32,33,34]. Figure 9 shows the properties of commonly used fiber materials.

**Figure 10:**
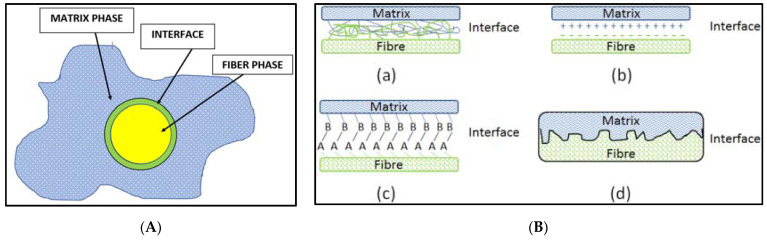
(**A**) Fiber-matrix composite material structure. (**B**) Fiber-matrix interfacial bonding mechanisms: (**a**) molecular entanglement following interdiffusion, (**b**) electrostatic adhesion, (**c**) chemical bonding, and (**d**) mechanical interlocking [39] (reprinted with kind permission from Elsevier).

In order to bond matrix and fiber together, a bonding agent is utilized at the interface of both as shown in the Figure 10A [36]. To perform adequately, a composite material should be bonded properly at the interface region [37]. The interface has an important role, as it transfers a load from the matrix to the reinforcements. Better bonding at the interface results in higher stiffness and strength of the resulting composite [38]. 

Figure 10B shows the different types of interfacial bonding mechanisms between fiber and matrix. Interfacial adhesion between the fiber and matrix interface is the critical factor that significantly dictates the stress transfer between matrix and fibers. In the literature [40], different mechanisms for fiber-matrix bonding have been reported such as inter-diffusion, electro-static adhesion, chemical reaction, and mechanical interlocking as reported in the Figure 10B. These mechanisms contribute in combination to make the interfacial adhesion between layers. When fiber and matrix materials came into contact at the molecular level, a diffusion process occurs due to the Van der Waal interaction and hydrogen bonding. This process is controlled by the good wetting behavior between fiber and matrix materials and the degree of diffusion. The degree of diffusion is mainly dependent on the chemical compatibility of the fiber and matrix materials. In electrostatic adhesion mechanism, the bonding between fiber and matrix surfaces is the result of anion and cation formation at the surfaces that results in adhesion. In the chemical bonding mechanism, surface chemistry has a controlling influence. Mechanical interlocking is the result of penetration and locking of peaks and valleys between fiber and matrix materials. 

Poor bonding at the interface provides lower strength and stiffness. Failure mechanisms are also characterized by fracture at the interface or away from interface, namely, adhesive and cohesive fractures, respectively. 

## 4. 3D Printing of FRP Composites

**Figure 11:**
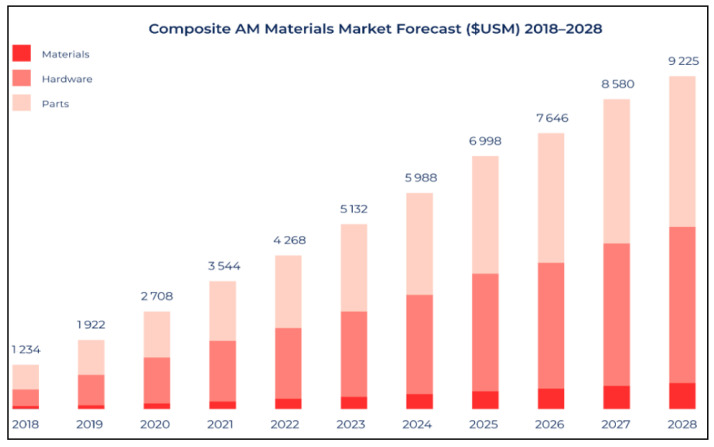
Growth in additive manufacturing of composites [43].

It has been observed that conventional FRP composite fabrication methods are expensive, time consuming, and rigid to design modifications [41]. 3D printing is an additive manufacturing technique; in 3D printing, layer-by-layer components are fabricated instead of cutting or molding the material. 3D printing can produce complex and customized products with a short delivery time, lower production cost, and lower material consumption. Currently, 3D printing is used in applications, such as individual production, complex products manufacturing, on-demand manufacturing, new business models, new services, and decentralized products. 3D printing is without a doubt a technology that will rule the future and will represent new stage of smart manufacturing [42]. The global forecast for growth in additive manufacturing of composites is predicted to be a 10 billion USD overall opportunity as shown in Figure 11 [43]. The process of fabricating CFRP in a cost-effective way is under investigation. It is also predicted that 3D-printing technologies will bring positive changes in the cost, energy, and emissions throughout the life cycle of parts [44]. Another comparative study showed that cumulative energy demand was reduced by 41–64% using 3D-printed polymeric materials [45].

The most popular type of 3D-printing technologies is FDM. Other techniques that are used in 3D printing are selective laser sintering (SLS), laminated object manufacturing (LOM), PolyJet, digital light processing (DLP), selective deposition lamination (SDL), and electron beam melting (EBM). The materials used in FDM 3D printing are polymer filaments (PLA, TPU, ABS). The strength of 3D-printed materials is discussed in a later part of the manuscript. 

Currently, the strength of 3D-printed components is not up to the industry’s requirements, especially load-bearing parts and fully functional industry parts [46,47]. It is because of the lower end of the polymeric material system, commonly known as commodity thermoplastics, mainly used in 3D printing applications but have limited functionality based on load bearing capacity. Thus, if CFRPs and 3D printing are combined together, we can fabricate the best products that are light weight, high performance, complex in structure, and have good prospects in future industries [48,49]. Therefore, if we can combine CFRPs and 3D printing together and take advantage of both, we can produce light-weight and high-performance components more efficiently with more complex structures, which will have very good prospects in future manufacturing industries [50]. CFRPs have mechanical properties that can improve the life span of the products manufactured. These products are then used in the aerospace industry and small manufacturing sector so that light-weight products with durability can be manufactured [51,52].

### 4.1. Fused Deposition Modeling (FDM)

**Figure 12:**
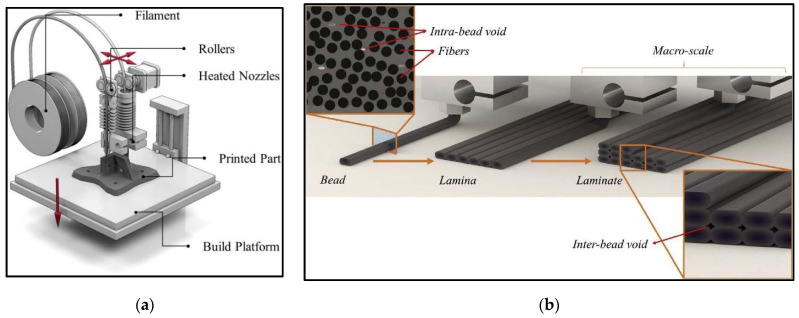
(**a**) Schematic illustration of conventional FDM Processes; (**b**) structural illustration of the FRP-printed part [54] (reprinted with kind permission from Elsevier).

FDM is a 3D-printing method based on material extrusion. In this process, extruded heated material comes out of nozzle and adds, layer by layer, to result in a printed composite part [53]. Filaments are made up of thermal plastics. The FDM process consists of the deposition of two materials: one to build the actual component and the other with disposable structural support. Filament is available in the form of spools and fed to the extrusion head. The extrusion head has a temperature controlling mechanism that facilitates heating of filament and conversion into a semi-liquid state. Figure 12a represents the schematic illustration of the FDM process. A spool of filament is utilized to melt first in the nozzle and then print the material in a layer-by-layer sequence to generate required geometric feature. To print the FRP composites using the FDM printing method, there are two methodologies. Figure 12b shows the structural representation of the FRP printed product. It shows the nomenclature in terms of bead, lamina, and laminate and intra-bead and inter-bead voids. 

### 4.2. Short Fiber-Polymer Composites Using FDM 

**Figure 13:**
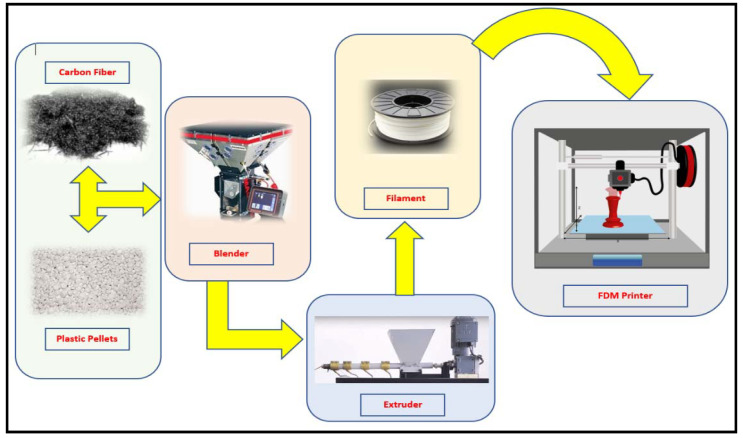
Schematic illustration of material flow in the first FRP printing strategy using a mixture of short fibers and polymer.

In the first method, a filament is a mixture of short fibers and polymer and printing happens using the conventional FDM-based printing process. Figure 13 shows the schematic illustration of the material flow in the FDM printing of the FRPs. The figure shows that CF and plastic pellets are blended together in the blender and extruded in the form of filament. The prepared CF-polymer filament is then utilized in the conventional FDM printer for printing purpose. 

### 4.3. Continuous Fiber-Polymer Composites Using FDM 

**Figure 14:**
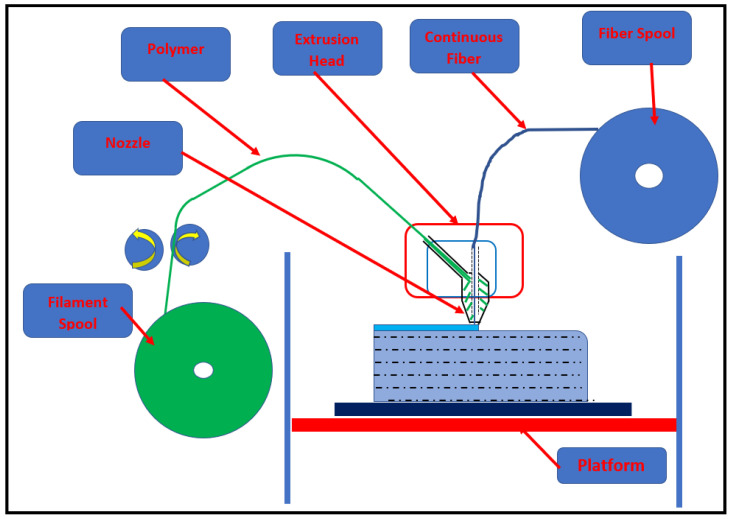
Schematic illustration of the second FRP printing strategy using continuous fibers and polymer (redrawn from [59]).

In the second approach, fiber is in a continuous form and mixed with polymeric matrix to print functional parts. The literature [55,56,57] reveals that there are three different options to incorporate continuous fiber in the matrix. These methods are differentiated in terms of timing and location of fiber-matrix mixing. In the first simple approach, prefabricated composite filaments are utilized to print the part using conventionally available FDM printers. It is referred to as a simple approach because it requires little change from the conventional FDM setup. In the second approach, the fiber and matrix are separated prior to reaching the print head, and they mix in the printing head that makes the mixing flexible. However, it comes with a challenging printing head setup. Air inclusion should be avoided by precise control during the fiber infiltration process [55]. Figure 14 represents the second type of printing arrangement. In the third case, fiber is deposited directly onto the polymeric component using separate mechanisms. Fiber impregnation is critical in this case, and inappropriate fiber deposition causes defects in the 3D-printed FRP composites. However, temperature-controlled post-processing can increase the strength significantly [58]. Table 2 shows different types of FDM-based FRP printing methods.

## 5. Mechanical Properties of Fiber-Reinforced Polymer Composites

The performance of any material is assessed from the mechanical properties. Mechanical character of material is judged by its yield strength, tensile strength, modulus of elasticity, and flexural strength. These properties are important in determining the performance of materials. Several studies were conducted to analyze the mechanical character of both short and long fiber 3D-printed FRP composites. 

### 5.1. Elastic Modulus and Strength

**Figure 15:**
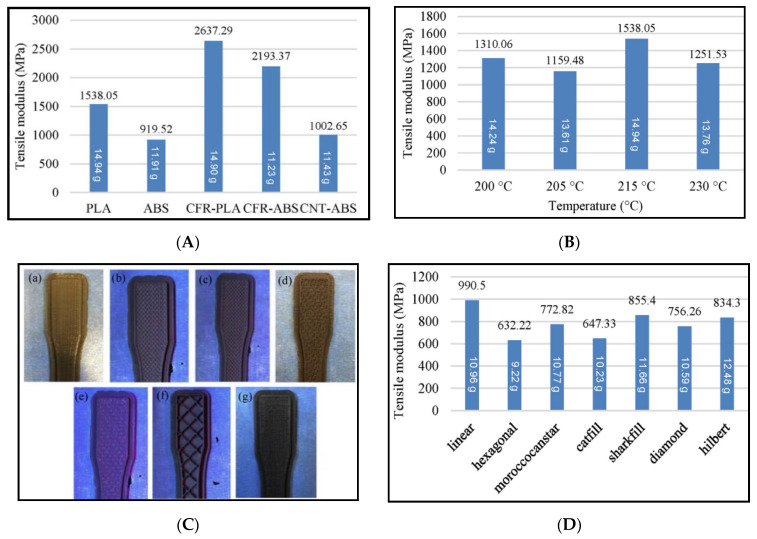
(**A**) Modulus of five different 3D printed materials. (**B**) Modulus of PLA at different nozzle temperatures. (**C**) PLA sample with infill patterns: (**a**) linear, (**b**) hexagonal, (**c**) moroccanstar, (**d**) catfill, (**e**) sharkfill, (**f**) diamond, and (**g**) Hilbert. (**D**) Modulus of PLA@50% infill density under different infill patterns (available under Creative Commons license [63]).

Ning et al. [5] investigated if mixing carbon fiber in different content percentages and fiber lengths could improve the mechanical properties in comparison to pure thermoplastic, which was ABS plastic in the study. The study incorporated different fiber content, such as 3%, 5%, 7.5%, 10%, and 15%. Two different fiber lengths of 100 and 150 μm were investigated. The study revealed that highest tensile strength was observed for the fiber content between 5% and 7.5%. For higher fiber content, tensile strength was reduced by approximately 10%. Higher levels of porosity were observed for fiber contents of 10% and linked with low strength. The 3D printing of FRP composites showed that by increasing the carbon fiber content in the product, it resulted in large void areas, and these voids negatively impacted on the tensile strength of the material [5]. Tekinalp et al. [60] mixed chopped short carbon fibers in the ABS matrix. The study revealed that the tensile strength and modulus was improved significantly when compared with the conventional compression molded composite samples. It was observed that 3D printed samples showed 115% increase in tensile strength, and approximately 700% increase in the modulus. Karsli and Aytac [61] prepared FRP composite by mixing short carbon fiber in polyamide 6 (PA6) matrix. The study investigated the mechanical properties of the prepared FRP by considering the fiber length and fiber content as the main parameters. Increasing fiber content resulted in better strength, modulus, and hardness values at the expense of ductility. Zhong et al. [62] mixed short glass fibers in the ABS matrix and improved the strength significantly. Abeykoon et al. [63] investigated the performance of five commercially available printing materials such as polylactic acid (PLA), acrylonitrile butadiene styrene (ABS), carbon fiber-reinforced PLA (CFR-PLA), carbon fiber-reinforced ABS (CFR-ABS), and carbon nanotube-reinforced ABS (CNT-ABS). The study aimed to investigate the effects of infill pattern, infill density, and infill speeds. It was observed in the study that higher infill density increased the modulus. Out of the different infill patterns, linear provided the highest modulus as shown in Figure 15C,D. The best performance of linear pattern was attributed with the layer arrangement with lowest pores and higher bonding between layers. The strongest material among the five materials was CFR-PLA as shown in Figure 15A. A nozzle temperature of 215 °C was found to be appropriate for PLA as shown in Figure 15B. Table 3 shows the summary of few studies with short fiber reinforcement.

**Figure 16:**
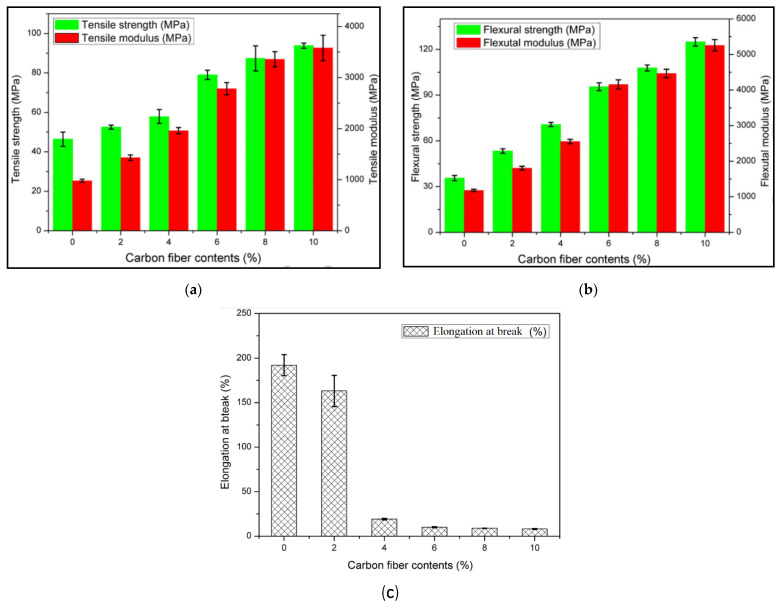
Effect of increasing fiber content % wt: (**a**) tensile modulus and strength; (**b**) flexural modulus and strength; (**c**) elongation at break (%) [66] (reprinted with kind permission from Elsevier).

The weak bond between the polymer matrix and the carbon fiber highly impacts the mechanical properties of the material. In the same case of weak bonds, however, the tensile strength and flexural strength could be improved by surface treatment with methylene dichloride and PLA trimmings [4]. Several studies were conducted on continuous fiber reinforced printing as well. Li et al. [64] produced 3D printed FRP composites by using PLA matrix and continuous carbon fiber filament. It was observed that desirable interface bonding was achieved using 3D printing method, as a result tensile strength was improved by 13.8% and flexural strength was improved by 164%. Yang et al. [65] fabricated the composite sample using a 10% fiber part of continuous carbon fiber (CCF) and ABS polymer using an additive manufacturing technique. The samples made proved to have an improved flexural strength of 127 MPa and a tensile strength of 147 MPa. These results were close in nature to the injection moldering made CCF/ABS composites. Liao et al. [66] developed FRP composite using continuous carbon fiber in polyamide 12 (PA12) matrix. The study revealed that better performance was observed for the carbon content of 10% wt as shown in the Figure 16a–c. Heidari-Rarani et al. [57] investigated the 3D printing of continuous fiber-based PLA matrix. The study aimed to investigate the influence of printing temperature, printing speed, fiber tension, and fiber surface conditions on tensile and bending properties. In addition, the study explored the development and designing of a user-friendly extruder that can be used with conventional FDM printers. The study utilized embedding on the workpiece method of continuous fiber printing method. The experimental findings revealed that tensile and bending strengths were improved by 35% and 108% when PLA matrix was printed with the continuous carbon fiber. Table 4 provides a brief summary of some studies with continuous fiber FDM 3D printing method.

### 5.2. Fatigue Strength

**Figure 17:**
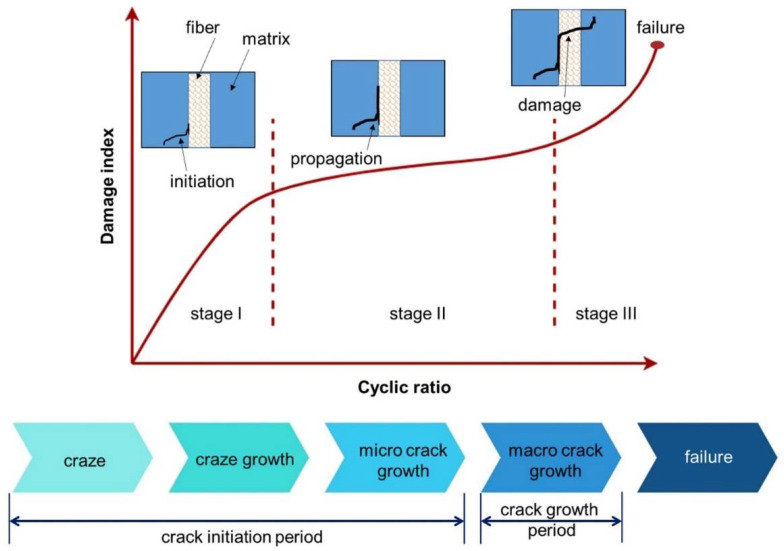
Phases of failure in fiber-polymer composites (available under Creative Commons license [67]).

Fatigue strength is considered an important criterion that contributes significantly towards the functionality of the 3D printed component. Shanmugam et al. [67] provided a detailed study to reveal the fatigue strength of 3D-printed polymeric material, 3D-printed polymeric composites, and 3D-printed cellular materials. The fatigue behavior of 3D-printed composite materials is very complex due to the anisotropic nature and layer by layer adhesion. Figure 17 represents the failure mechanism in the fiber-polymer composites in the form of three phases. The initial phase of failure is linked with the fiber matrix debonding in the regions where poor bonding is present. The reasons for poor bonding are reported to be fiber misalignment, matrix richness and poor surface conditions such as pores or voids. In the second phase, delamination occurs between fiber and matrix materials. In the final stage, crack propagation occurs at the fiber, and localized fractures occur [68]. Travieso-Rodriguez et al. [69] utilized the Taguchi design of experiments to study the fatigue performance of wood-PLA-based composite material. The study revealed the that 75% infill density, a 0.7 mm nozzle diameter, and a 0.4 mm layer height was the optimal combination. Printing velocity was found to have no significant influence on the performance. The lower endurance limit was found to be 17.9 MPa. Fatigue performance of the 3D-printed fiber-reinforced composites was highly dependent on the fiber volume fraction. Higher fatigue strength is achieved by higher volume fraction. Higher fiber orientation can result in poor fatigue performance. Higher fiber orientation can result in poor fatigue performance. It was also found that fatigue life was dependent on the stress ratio. A higher stress ratio provides a low fatigue life [68].

### 5.3. Creep Strength

**Figure 18:**
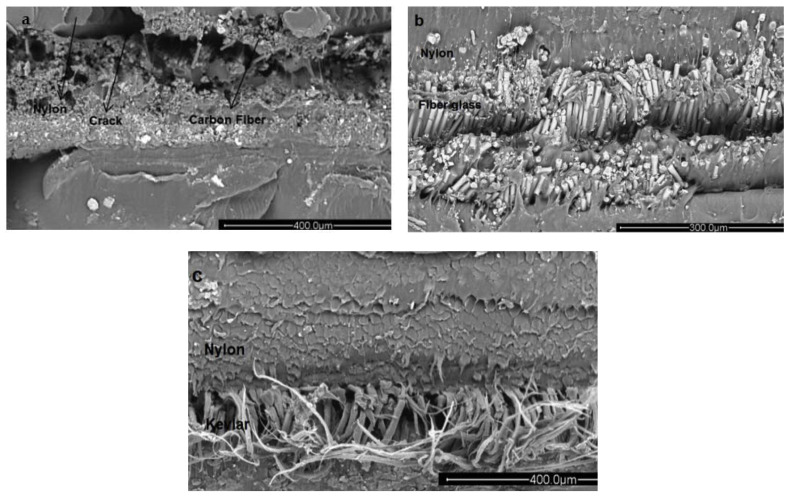
SEM images of creep fractured samples: (**a**) nylon-carbon fiber; (**b**) nylon-fiber glass; (**c**) nylon-Kelvar [72] (reprinted with kind permission from Elsevier).

Creep performance of 3D-printed FRP materials is an important parameter towards the reliable functionality of a product. It is rare in the literature for the creep performance of 3D-printed FRP materials to be investigated. Waseem et al. [70] performed a study where creep performance of 3D printed PLA was investigated using multiple response optimization. The study utilized different performance parameters such as infill pattern, layer height and infill percentages. Three levels of each parameter were investigated such as layer height which varied from 0.1–0.3 mm; infill patterns were linear; hexagonal, diamond, and infill percentages varied from 10–100%. The optimal condition of the hexagonal pattern, a 0.1 mm layer height, and 100% infill density was recommended in the study. Zhang et al. [71] investigated the creep performance of 3D printed ABS materials. The study also investigated creep performance under different printing orientations. The study revealed that a 90-degree printing orientation provided the highest creep resistance. Mohammadizadeh et al. [72] investigated the tensile, fatigue and creep performance of 3D printed fiber polymer composites. The samples were prepared using nylon and fibers of Kevlar, carbon fiber, and fiber glass. Higher void formation was observed in the SEM observed for creep. Higher creep strains were observed when the temperature increased to the glass transition temperature due to the higher macromolecular mobility in the polymeric chains. Figure 18 the scanning electron micrographs of fractured samples in creep testing. 

## 6. Complexities in FRP Composite 3D Printing Using FDM

**Figure 19:**
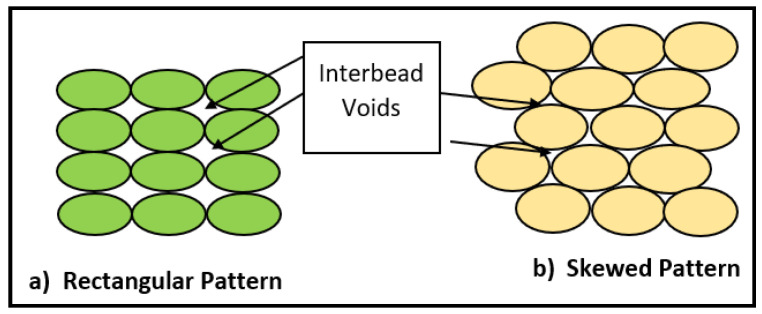
Schematic illustration of interbead void formation under different printing strategies (**a**) Rectangular pattern (**b**) Skewed pattern (redrawn from [78]).

**Figure 20:**
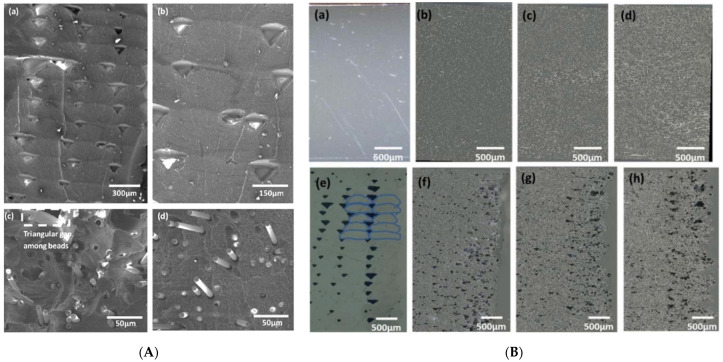
(**A**) Fractography of (**a**,**b**) conventional FDM-ABS (**c**) FDM printed with 10% wt. carbon fiber and (**d**) 10% wt. carbon fiber compression molded [60]. (**B**) Short fiber-based composites by compression molding (**a**–**d**), FDM-printed short fiber composites (**e**–**h**) with the fiber volume fraction changing from unfilled to 30 wt.% with a 10% increment [60] (reprinted with kind permission from Elsevier).

Generally, the traditional way of molding carbon fiber is a time taking process and costly as well. But integrating it with additive manufacturing helps reduce cost and save time as well, increasing efficiency in case of complex shapes especially [3,47,51,52,53,73]. For short fiber reinforced composites, carbon fibers (in short length segments) are mixed with other thermoplastics for printing. They are extruded to obtain a filament which can be used in 3D printer to manufacture various shapers and parts. Increasing the carbon fiber content increases the tensile strength and hardness but the effect of reinforcement for short fibers is less than long fibers. It was also observed that fibers have low wettability when combined with the thermoplastics resulting in poor behavior and also it makes fiber loading in filament problematic. The performance of 3D printed parts is linked with interlayer bonding between the consecutive layers. The melt dynamics of plastic are linked with the temperature and viscosity behavior during extrusion process [74,75]. The literature revealed that voids form during the printing of adjacent layers, and it has a controlling influence on the strength-related character of the printed FRP composite materials [14,76,77]. Different printing strategies can be adopted to decrease the void density in the printed samples as shown in the Figure 19. Reducing the interbead voids in the sample, increases the load bearing capacity of the printed sample. Figure 20A shows fractographs of neat ABS and CF-ABS under FDM and Conventional compression molded samples. It can be observed that fibers are bulging out showing poor interfacial adhesion between fiber and matrix. It can also be observed that larger pores were present in the FDM (ABS/CF) as compared to the compression molded sample. Figure 20B shows that as the CF is added to the neat ABS, the triangular channel between beads is reduced. It is associated with a reduction in die-swell and improvement in the thermal conductivity due to the CF.

**Figure 21:**
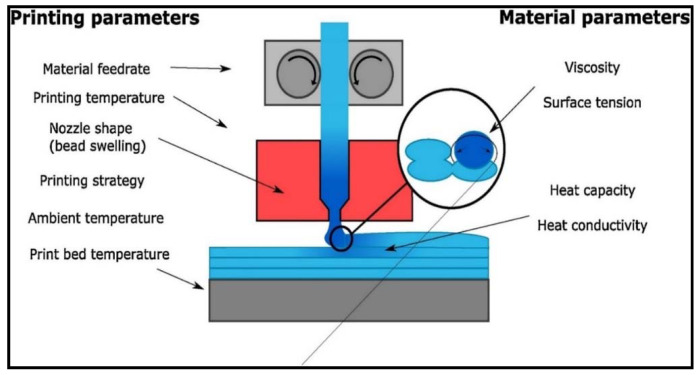
Influencing parameters to attain best possible sintering between different polymer layers (available under a Creative Commons license [81]).

The functionality of 3D printed FRP composites is strongly associated with the sintering of polymeric materials. Sintering in 3D printing is controlled through temperature and surface contact. Temperature is important because it governs the flow properties by influencing the surface tension and viscosity of the molten polymeric material. Heat distribution in the printed layers is controlled by the thermal conductivity and heat capacity [79,80]. The whole process becomes more complex due to the involvement of fiber content and dependency of material parameters with respect to the printing parameters. Figure 21 shows the influencing parameters to get optimal level of sintering between different polymeric layers.

It has also been observed that stiffness improves but strength is not improved significantly. This is due to the fiber pull out phenomenon that takes place before fiber fracture. In addition, increasing carbon fibers lead to larger areas of void which starts affecting the tensile strength negatively. Moreover, the resulting composite starts losing ductility and yield strength. Poor bonding between other materials and carbon fiber can significantly affect mechanical characteristics. Pure continuous carbon fiber when 3D printed has a better performance, but its major weaknesses are longer processing times and they cost more [37].

## 7. Industrial Developments to Print FRP Using FDM

According to a recent study, it is expected that the global market for 3D printing is projected to grow from USD 12.6 billion (in 2021) to USD 34.8 billion (by 2026) at a 22.5% compound annual growth rate (CAGR). 3D printing of composites is still in an emerging stage. But many industrial technologies, including defense, automotive, and aerospace, possess huge opportunities for 3D CFRP printing. This has many advantages such as reducing part manufacturing time and waste, achieving intricate geometries, and no expensive tooling is required. Currently, 3D printing is being used for the manufacturing of tools made of composites and the composite prototype parts. But the continuous advancement in 3D printing of FRP and the increasing interest of companies in additive manufacturing of composites will take the market to new heights. Many big names are at the forefront of using FRP-based 3D printing technology. For example, in 2017, Stratasys, an additive manufacturing company, launched a nylon-filled carbon fiber product for rapid proto-typing, composite tools, and high-end applications instead of using metals.

**Figure 22:**
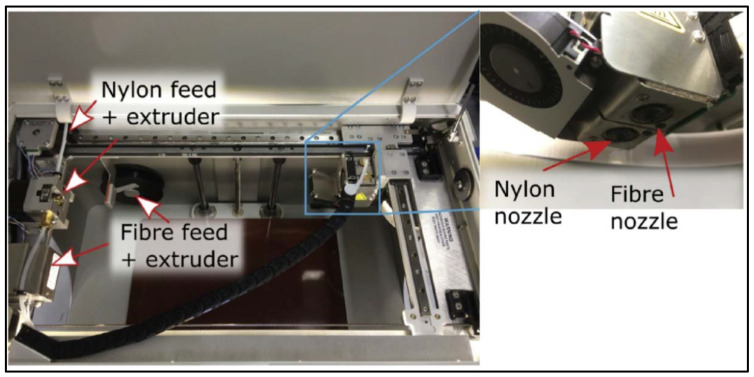
Dual nozzle system on MarkOne printer (available under a Creative Commons license [81]).

3D composite printing plays an imperative role in meeting customer needs by manufacturing various parts utilizing less time and reducing wastage. The market is segmented based on composite type (continuous fibers or discontinuous fibers), reinforcement type (e.g., carbon fiber or glass fiber) or the technology type (e.g., extrusion, powder bed fusion etc.). Also, carbon fiber reinforcement is preferable due to the wide variety of applications and advantages such as high strength, low weight and resistance to fatigue and corrosion. It is also high in demand in major industries including aerospace, automotive, defense and medical sectors due to the biocompatibility and light-weight parts for structural applications to improve the fuel efficiency and reducing carbon emissions. Based on regions, North America is the most dominant market for 3D printing of composites while the European market contributes as the 2nd largest. Some of the well-known names for the 3D printed composite parts in market include Stratasys Ltd., Cincinnati Incorporated, Arevo Labs, Mark Forged, 3D Systems Corporation, Inc., Graphite Additive Manufacturing Limited, and CRP Group. For example, the Figure 22 below shows a MarkOne printer with dual nozzles for nylon and fiber injection [81].

Additive manufacturing or 3D printing of polymer fiber composites, such as carbon fiber, is a vigorous manufacturing model. These composites provide flexibility in structure and enhanced mechanical properties. By reading the papers added in the review, one thing observed is that the FDM is the most commonly used additive manufacturing technique for the preparation of carbon fiber-reinforced polymers. Currently, FDM compatible thermoplastics are limited to amorphous polymers and polymers with low crystalline level acrylonitrile butadiene styrene (ABS) and polylactic acid (PLA) [17,31]. The study’s focus is primarily on the mechanical properties of FRP composites fabricated using FDM techniques of 3D printing. The major usage of carbon fiber in products fabrication is because of the high-strength-to-weight-ratio and light weightiness of the material. The literate reviewed also reported the usage of varied thermoplastic polymers and short carbon fiber of 0.1 mm, this matrix is reinforced using 3D printing by using a slow extrusion process. Using short fibers proved to give improved strength of printed products. It has been reported that average fiber length reduces as the fiber loading increases during FDM. During the mixing of fibers with resin, the fiber length reduced drastically due to the following reasons such as contact of mixing instrument, resin contact, and contact with other fibers [35,36]. 

## 8. Data-Driven Based Machine Learning (ML) Approaches

**Figure 23:**
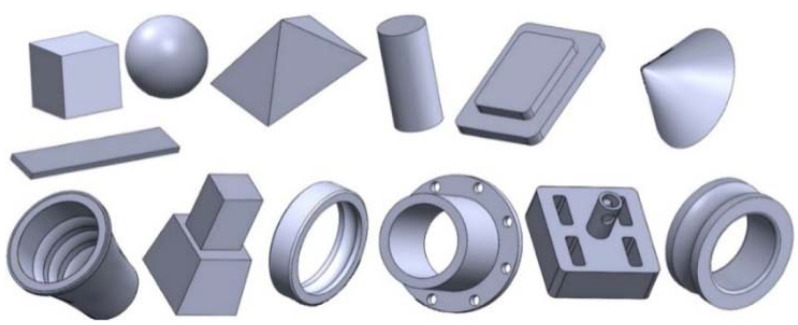
Data prepared to train a machine learning model [88] (reprinted with kind permission from Elsevier).

**Figure 24:**
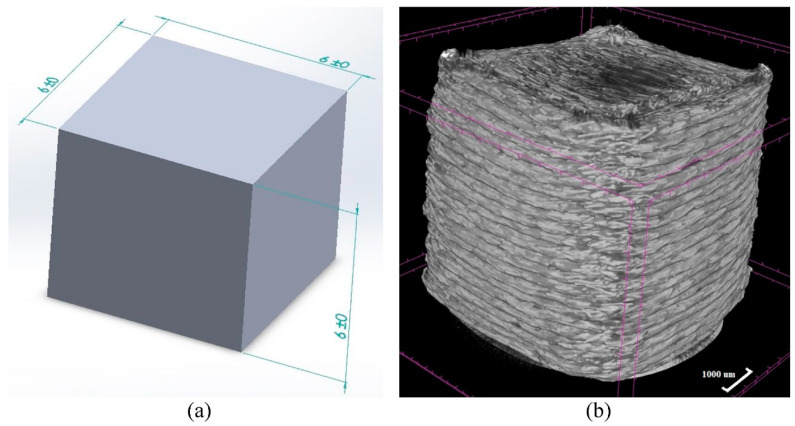
(**a**) CAD model with dimensions. (**b**) Micro-level CT scan of the 3D printed part [89] (reprinted with kind permission from Elsevier).

There is a lot of attention given to artificial intelligence (AI) and machining learning (ML)-based data-driven approaches these days. ML approaches are based on recognizing the patterns from complex data. Goh et al. [82] provided a very detailed review of the ML approaches (supervised, semi-supervised, reinforced, and unsupervised) with respect to the 3D printing technologies. Charalampous et al. [83] conducted a study using regression-based machine learning approach to investigate the deviations between CAD model and the actual part. The study also discussed strategies to provide compensation related to the FDM process. Noriega et al. [84] conducted a study where an artificial neural network (ANN)-based algorithm was used to study the dimensional accuracy of FDM printed parts. The study revealed that 50% and 30% of dimensional errors were reduced for external and internal features using the proposed optimization approach. Vahabli and Rahmati [85] conducted a study using radial basis function neural networks (RBFNNs) to predict the surface finish of FDM printed parts. Optimization was performed using imperialistic competitive algorithm. The study revealed an error percentage of 7.11–3.64% for both models. Delli and Chang [86] provided a methodology to automatically monitor the 3D printed products using a machine learning approach, namely, support vector machine (SVM). Rayegani and Onwubolu [87] correlated the FDM process parameters with product strength using group method of data handling. The study developed a mathematical model by using the controlling parameters of orientation, raster angle, raster width and air gap. The results of this work were very practical and encouraging and can be easily implemented in the industry. Hooda et al. [88] utilized AI data-driven approach to reduce manufacturing time and cost of the product. The study discussed the deposition angle optimization with respect to the product geometry. Prediction accuracy of 94.57% was obtained by the proposed methodology. Figure 23 and Table 5 shows the parameters used in this work and the CAD models used to train the model. Yanamandra et al. [89] revealed an important application of reverse engineering of 3D printed composite part using imaging and machine learning assisted approach. The study analyzed the microstructure using the machine learning approach and even the tool was reconstructed. Figure 24a,b shows the CAD model and micro-level CT scans of layers. The approach revealed an error of only 0.33%. Jiang et al. [90] utilized back propagation neural network (BPNN) to study the parameters involved in unsupported bridge length for 3D printed sample component. It has been revealed that BPNN correctly provide the optimal longest distance between point that can hang unsupported. 

## 9. Conclusions

After reviewing the literature, we can say that new and enhanced mechanical properties of materials with light-weight composition and greater flexibility can be achieved using various 3D printing techniques with carbon-fiber reinforced polymers. The 3D printing of carbon-fiber reinforced polymers is preferred to be performed using FDM technology. The material used in this type are separate carbon fiber filaments or saturated carbon fiber filaments. The FDM printer is modified to achieve co-extrusion, cutting, and fixed-shape properties. LOM type printers are using carbon fiber-impregnated films for product manufacturing; this technique is not fully developed yet. New technologies for automated fiber placement and laser tape-assisted winding are close in nature to additive manufacturing, and the concept of using these looks promising in applications of continuous carbon fiber-reinforced polymers. The continuous carbon fiber-reinforced thermoplastics by 3D printing is a novel concept due to the high efficiency and low-cost potential. 

The continuous fiber placement for 3D-printed CFRP composites requires new algorithms so that accurate placement of the fiber can be made possible. There is a research gap that exists in understanding the long-term performance of CFRPs products fabrication using 3D printing technology. FRP materials are used in product fabrication to attain better strength. The material selection is the critical criteria for predicting the strength of the CFRPs in the long term. Moreover, the CFRP sheets encounter the problems of fiber rupture, micro-cracks in the structure, and the resin de-bounding with the passage of the time strength. For future research, studies should be conducted on the use of recycled carbon fibers, so that cheaper and less energy consuming products can be fabricated. These recycled carbon fibers will be helpful in reducing the environmental and financial impacts of additive manufacturing of CFRPs using virgin fibers. Another area for future research work is to study the physical and mechanical properties of carbon fiber reinforced polymers from different aspects, different than the ductile strength and the flexural properties. The results of these studies will be helpful in analyzing the potential of CFRPs fabricated using additive manufacturing and will open ways to new markets. One more field highlighted is to investigate the long-term usage impact and wear and tear in the structure of 3D-printed carbon fiber-reinforced polymers.

In order to enhance the performance of 3D printed FRP materials, there is a need improve the interfacial bonding between the fiber and matrix to improve the functional performance of the 3D printed materials. Limiting the porosities in the 3D printed FRP materials can also result in the improved performance. Parameters, such as fiber volume fraction, atmospheric environment, cooling rate, temperature of the nozzle, and printing speed. can be optimized to limit porosities.

Fatigue performance of the 3D-printed fiber-reinforced composites is highly dependent on the fiber volume fraction. Higher fatigue strength is achieved by higher volume fraction. Higher fiber orientation can result in poor fatigue performance. It was also found that fatigue life was dependent on the stress ratio. A higher stress ratio provides a low fatigue life. Higher creep strains were observed when the temperature increased to the glass transition temperature due to the higher macromolecular mobility in the polymeric chains.

Automated quality inspection of the 3D printed part is an emerging area these days. It has been seen that artificial intelligence-based machine learning approaches have good potential in this application. However, the majority of work is performed on conventional FDM printing for polymers only. There is a need to enhance this area for potential quality inspection of FDM-printed fiber-reinforced composites as well. Machine learning was also identified as a power tool to reengineer the microstructure of 3D-printed composite product. Machine learning based approaches can also be utilized efficiently to optimize the hanging lengths of the 3D printed product for optimal design solutions. 

## Figures and Tables

**Table 1:** Different properties and industrial applications of matrix materials [30,31,32,33,34].

Sr. No.	Matrix Material	Properties	Major Industrial Sector
1	Polyether sulfone	Flame resistance	Automotive
2	Polyphenylene sulfide	Chemical and high temperature resistance	Electrical
3	Polysulfone	Low moisture absorption and high creep strength	Marine
4	Polyethylene (PE)	Corrosion resistance	Piping construction
5	Polypropylene (PP)	Chemical resistance	Packaging
6	Polylactic acid (PLA)	Biodegradable nature	Biomedical
7	Polyurethane (PU)	Wear resistance and waterproof	Structural
8	Natural Rubber	Low density	Automotive
9	Epoxy Resin	High strength	Automotive and aerospace
10	Polyester	Durable and resistance to water	Structural

**Table 2:** 3D printing method.

	Pre-Embedded Composite Filament	Embedding in Print Head	Embedding on Component
Advantages	- Improved handling while printing due to the prefabrication of filament. - Easy to incorporate in a conventional FDM printer.	- Ability to print both pure plastic and plastic–fiber mixed parts. - Easy to vary fiber volume ratio.	- Process is more versatile and different fibers and matrices materials can be added.
Disadvantages	- Rigid in terms of material mixture, as it provides a fixed fiber–volume ratio.	- Requires special printing head with precise control over mixing and air inclusion.	- Adjustment of fiber orientation requires additional complex machine capabilities.

**Table 3:** Summary of the literature on short fiber reinforcement.

Source	Matrix	Reinforcement	Important Findings
Ning et al. [5]	ABS	Carbon fiber powder (100 μm, 150 μm)	- Tensile strength of 42 MPa was highest for 5% wt fiber and lowest 34 MPa for by 10% wt. - 100 μm fiber length specimen showed higher ductility and toughness with respect to 150 μm.
Tekinalp et al. [60]	ABS	Short carbon fiber (0.2–0.4 mm, after mixing: 0.26 mm)	- 3D printed composite samples showed 115% higher tensile strength and 700% higher modulus when compared with the conventional compression molded composites.
Karsli and Aytac [61]	Polyamide 6	Short carbon fiber (0–50 μm)	- Increasing fiber content improved strength, modulus, and hardness. - However, increasing fiber content resulted in lower strain at break value.
Zhong et al. [62]	ABS	Short glass fiber	- Strength of ABS was improved significantly at the expense of low hand ability and poor flexibility.
Abeykoon et al. [63]	Polylactic acid (PLA), acrylonitrile butadiene styrene (ABS), carbon fiber-reinforced PLA (CFR-PLA), carbon fiber-reinforced ABS (CFR-ABS), and carbon nanotube-reinforced ABS (CNT-ABS)		- To obtain desirable results, the printing speed and nozzle temperature should match. - Higher modulus was obtained for higher infill density. - Linear pattern provided highest modulus due to the lower number of spaces in the sample. - CFR-PLA was found to be the strongest material.

**Table 4:** Table displaying the matrix, type of fiber reinforcement, and type of printing technique.

Source	Matrix	Reinforcement	Important Findings
Li et al. [64]	PLA	Continuous carbon fiber	- 3D printed samples showed desirable bonding at the interface and increased tensile and flexural strengths by 13.8% and 164%, respectively.
Yang et al. [65]	ABS	Continuous carbon fiber	- 10% wt continuous fiber in ABS increased the tensile and flexural strengths to 127 MPa and 147 MPa.
Liao et al. [66]	Polyamide	Continuous carbon fiber (6–7 μm)	- Adding 10% wt. fiber content to PA 12 increased tensile strength, flexural strength, and modulus without affecting behavior.
Heidari-Rarani et al. [57]	PLA	Continuous carbon fiber	- The tensile and bending strengths were improved by 35% and 108% when PLA matrix was printed with the continuous carbon fiber.

**Table 5:** FDM parameters and parts based data [88].

Process Time (min)	Weight Material (g)	Length of Material Wire (m)	Orientation	Deposition Angle
77	6	2.43	Flat	0
107	7	2.57	Flat	15
138	5	2.07	Upright	60
158	6	2.22	Edge	15
146	6	2.21	Edge	30
84	6	2.26	Flat	75
88	6	2.31	Flat	90
76	6	2.43	Upright	0
110	7	2.55	Upright	15

## Data Availability

Not applicable.
